# Symbolic estrangement or symbolic integration of numerals with quantities: Methodological pitfalls and a possible solution

**DOI:** 10.1371/journal.pone.0200808

**Published:** 2018-07-16

**Authors:** Mila Marinova, Delphine Sasanguie, Bert Reynvoet

**Affiliations:** 1 Brain and Cognition, Faculty of Psychology and Educational Sciences, KU Leuven, Leuven, Belgium; 2 Faculty of Psychology and Educational Sciences, KU Leuven @Kulak, Kortrijk, Belgium; University of Melbourne, AUSTRALIA

## Abstract

Previous studies, which examined whether symbolic and non-symbolic quantity representations are processed by two independent systems or by one common system, reached contradicting findings, possibly due to methodological differences. Indeed, some researchers advocate the two systems approach, based on the presence of notation-specific switch cost in conditions where adults have to compare pairs of symbolic and non-symbolic quantities, in combination with the absence of such a cost in conditions containing quantities of the same notation. However, other researchers used matching instructions, and reported a facilitation in the mixed notation conditions, suggesting that the two systems are automatically integrated. In the current study, we conducted three experiments, in which we examined the existence of two separate quantity systems, but we used various experimental manipulations (e.g., task instructions, presentation order) to unravel the previous inconsistent findings. In Experiment 1, we investigated the role of task instructions by presenting participants with pure and mixed notation trials with both comparison and matching tasks. In Experiment 2, we tested the role of blocked and randomized presentation order for the pure and mixed trials. Our data showed that cost for switching between the symbolic and non-symbolic quantities is present, but is prone to a certain methodological drawback: when the differences between the processing times for two sequentially presented stimuli of different notations are not taken into account, this masks the cost for switching between the two systems. To overcome this problem, in Experiment 3 we used an audio-visual paradigm. Overall, our results provide further evidence for the existence of distinct quantity representations, independently of task instructions or presentation order. Additionally, considering this methodological pitfall we argue that the audio-visual paradigm is better suited when investigating the integration between symbolic and non- symbolic quantities.

## Introduction

For the past thirty years, the research domain of numerical cognition has been dominated by the view that symbolic numerals (e.g., Arabic numerals, number words) and non-symbolic numerosities (e.g., dot arrays) are represented by the same evolutionary evolved brain system [1], referred to as the ‘Approximate Number System’ or shortly ANS (e.g.,[2,3,4], but see [5]). Consequently, researchers have extensively argued that, in order to process exact symbolic quantities, a mapping process to the corresponding approximate non-symbolic quantity is required (e.g.,[6,7]). Arguments used in this regard are the presence of similar behavioral effects (e.g.,[8,9,10]), or commonly activated brain areas [11,12,13] when symbolic and non-symbolic stimuli are processed. By contrast however, there are also studies showing distinct performance on symbolic and non-symbolic tasks (e.g.,[14]) and format-dependent representations in the brain [15,16,17,18]. Consequently, these latter findings have challenged the dominant ‘mapping’ hypothesis, inducing several substantial theory revisions, and were the inspiration for an alternative proposal describing the existence of two separate systems for processing exact symbolic versus approximate non-symbolic numbers (e.g.,[19,5,20]).

To date however, only a limited number of studies have systematically examined the existence of two separate systems (e.g.,[21,22,23,14]), and the interpretations of their results are not entirely consistent. Lyons et al. [22] for instance instructed participants to compare pairs of quantities, presented in either pure symbolic format (Arabic digits–Ex. 2; number words–Ex. 3), pure non-symbolic format (dot arrays–Ex. 2 and 3), or mixed format trials (digits and dot arrays–Ex. 2; digits and number words–Ex. 3). The authors hypothesized that, if symbolic and non-symbolic quantities share common representations, then the performance in terms of reaction times and accuracy in the mixed digit-dot condition should be somewhere in between the performances on the pure digit and pure dot conditions. On the contrary, they observed worse performance (i.e., slower reaction times and more erroneous responses) in the mixed format trials, compared to the most difficult pure non-symbolic condition (i.e., the pure dot condition) and interpreted this result in terms of a ‘switch cost’ emerging from cognitive effort needed to link two *distinct* magnitude representations. In line with this reasoning, no such switch cost was present when comparing mixed and pure trials containing only symbolic elements (e.g., mixed digits and number words trials versus pure number word trials in Ex. 3), suggesting that number words and digits share a common symbolic representation.

Liu and colleagues [21] reached a different conclusion regarding the relation between symbolic and non-symbolic quantities, based on a presence of cross–format facilitation effect. In their study, participants had to judge if the presented stimulus was a number (e.g., “25”) or a letter pair (“QX”). Both numbers and the letter pairs were presented superimposed on task-irrelevant dot arrays. The numbers and the dot configurations were either matched or mismatched in terms of the quantity they represent (e.g., digit ‘25’ superimposed on 25 dots constitutes a ‘match’ trial, and digit ‘25’ superimposed on 39 dots–a ‘mismatch’ trial). After taking into account the typical underestimation of the dot arrays, the results showed that, although processing the dots was not a prerequisite for the task, a congruency effect was present in trials with numbers. Put otherwise, when the number and the dot array matched, participants responded more accurately and faster, compared to trials where the quantity information did not match. In contrast, no difference was observed with letter pairs in the performance for matched and mismatched trials. According to the interpretation of Liu et al. [21], these findings suggest that human adults integrate the symbolic and non-symbolic quantity representations automatically and effortlessly. In sum, the ways in which Lyons et al. [22] and Liu et al. [21] have interpreted their results and the implications from these interpretations with regard to the relation between symbolic and non-symbolic number systems are at odds with each other.

Possibly, these inconsistencies stem from the fact that these studies used conceptually different designs to investigate the same issue. Namely, Lyons et al. [14] based their design and interpretation of a ‘switch cost’ on studies investigating task switching, stating that if there is a cost for switching between pure and mixed trials, probably different representational systems are involved (e.g., [24]). The design of Liu et al. [21] is based on studies using Stroop–like methods, in which facilitation/interference effects from an irrelevant stimulus dimension on a relevant stimulus dimension are investigated (e.g., [25, 26]). However, the presence of a facilitation effect from irrelevant dots on the corresponding digit, as interpreted by Liu et al. [21], does not necessarily imply the existence of a symbolic integration and/or of one common representational system. The only thing that could be claimed is that there is an automatic associative relation between these two representations [26]. That is why, the findings of Liu et al. [21] could not be seamlessly reconciled with the previous studies claiming one common numerical system. Consequently, these conceptual differences between the studies of Lyons et al. [22] and Liu et al. [21] with respect to their designs and interpretations, make it difficult to draw a direct coherent conclusion about the relation between symbolic numbers and non–symbolic quantities.

Furthermore, the conceptually divergent starting points of Lyons et al. [22] and Liu et al. [21], described above, had as a consequence that both studies applied different tasks, which also might have contributed to diverse interpretations of their results.

For example, Lyons et al. [22] argued that the comparison task is well suited to examine the relation between symbolic and non-symbolic quantity representations because it urges participants to access “how much a given symbol represents explicitly” ([22] p.636). However, other researchers previously argued that the effects from the comparison task might result from decisional strategies (for details see [27]), and/or general sensorimotor transformations [28] and thus do not necessarily index the actual numerical magnitude representation. Consequently, other tasks have been proposed to be better suited for addressing the numerical magnitude representations. One such task is the numerical matching task, where participants have to judge if two magnitudes match or mismatch in their quantities [29,30]. This task is to some extent analogous to the “numberness task” used in Liu et al. [21]. Inasmuch as they took into account the actual dot quantity that participants perceived, the performance in this “numberness task” in fact reflects participants`abilities to implicitly match symbolic and non-symbolic quantities. Therefore, using different task instructions may tap into the magnitude representations to a different extent.

In conclusion, the conflicting interpretations from the two studies described above clearly show that there is no unified opinion in the literature regarding the question about the existence of a one common or two distinct number magnitude systems. Therefore, in the current study we investigated whether the degree to which evidence for distinct (e.g.,[22]) or for integrated (e.g.,[21]) symbolic and non-symbolic quantity representations is found, depends on the differences between the methodological manipulations used in these two studies. To this end, we conducted three experiments with adults. In Experiment 1, we replicated the experimental design of Experiments 2 and 3 of Lyons et al. [22], but now with participants given both comparison and matching instructions in separate conditions. This way we examined whether a switch cost was present when symbolic and non-symbolic number processing need to be integrated for a comparison decision (i.e., replication of Lyons et al. [22]) and whether these results can be generalized to matching instructions. In Experiment 2, we tested whether the presence of a switch cost depends on the order of the pure and mixed trials (i.e., blocked versus randomized). Given that these two experiments resulted in some pitfalls of the purely visual presentation, in Experiment 3 we overcame these problems by using audio–visual paradigm to examine the costs between pure and mixed trials.

## Experiment 1: Comparison and matching instructions

### Method

#### Participants

Thirty-six students from KU Leuven Kulak, aged between 17 and 28 (*M*_*age*_ = 18.88 years, *SD* = 2.24, 7 males), participated in this study for a small monetary compensation. The experimental protocol was approved by the Ethical Committee of the Faculty of Psychology and Educational Sciences of the University of Leuven (file number G−20160679). All participants gave written informed consent. All subjects had normal or corrected-to-normal vision. There were no outliers because none of the subjects performed at chance (≤50%) or were too slow/ fast (> 3*SD* above or below the group). (Data available in S1 Dataset).

#### Procedure, task instructions and stimuli

All participants performed both tasks subsequently with a small break in between (i.e., one with comparison and one with matching instructions) in six possible stimulus combinations: (1) digit–digit, (2) number word–number word, (3) dot–dot, (4) number word–digit, (5) digit–dot, and (6) number word–dot. All six combinations were randomly presented in both tasks. The presentation order for the stimuli in all mixed conditions was balanced (e.g., for the number word–digit condition half of the trials started with digit and half with number word, and same applied for the remaining mixed conditions). The order of the instructions was counterbalanced across participants.

E-prime 2.0 software (Psychology Software Tools, http://pstnet.com) controlled for the stimulus presentation and recording of the data. In the comparison instruction condition, participants had to decide which of the two sequentially presented quantities was larger. In the matching condition, they had to respond if the two sequentially presented stimuli were numerically the same or different. In both instruction conditions, the importance of speed as well as accuracy was emphasized. Participants each time gave their responses bimanually on an AZERTY keyboard, by pressing “a” with their left index finger, if the first stimulus was larger/if both were numerically equivalent, and “p” with their right index finger, if the second stimulus was larger/if both stimuli were numerically different.

Stimuli for the comparison instruction condition consisted of the quantities 1, 2, 3, 4 and 5, denoted as digits, number words or dot arrays in all possible combinations, excluding combinations containing the same numbers (e.g., ‘1–1’ or ‘1 –one’). Dot arrays were generated using the MATLAB script of Gebuis and Reynvoet [31] controlling for non-numerical cues (i.e., total surface, convex hull, density, dot size and circumference). All trials where stimulus combinations contained the quantities of 1 and/or 5 were considered filler trials, because these quantities were the smallest and largest in the range and therefore the judgments on these trials could be easily made on the basis of only one stimulus. Consequently, only trials containing quantities 2,3 and 4 were analyzed.

In case of the matching instruction condition, stimuli were the quantities 2, 3 and 4, denoted as digits, number words or dot arrays in all possible combinations, but now also including combinations containing the same values (e.g., ‘2–2’, or ‘•• – 2’). Here, all trials containing the same numerical value were considered fillers.

The task design was identical to Lyons et al. [22]. Stimuli appeared centrally on the screen in white font Times New Roman (size 44) on a black background. Each trial began with a 600ms fixation cross. Afterwards, the first stimulus appeared for 150ms, followed by a 700ms blank interval and the second stimulus for 150ms. Participants gave their responses during the blank screen that followed immediately after the second stimulus. The response was followed by a 1000ms intertrial interval, after which the next trial was presented. Prior to the actual experiment, participants were given 10 practice trials with feedback. No feedback was provided during the actual experiment. The filler-to-target trials ratio for the comparison condition was 2:1 (144 target and 86 fillers), whereas for matching it was 1:1 (144 target and 144 fillers).

### Results

First, mean accuracy scores and median reaction times (RT) on correct responses were submitted to a repeated-measures analysis of variance (ANOVA) with instruction (two levels: comparison vs. matching) and notation (six levels: digit–digit, number word–number word, dot–dot, number word–digit, digit–dot, number word–dot) as within-subject variables. Whenever the assumption of sphericity was violated, the Greenhouse–Geisser correction was reported. Mean accuracies and median reaction times (RT) per instruction and per notation are depicted in Table 1.

**Table 1:** Mean accuracies and median reaction times (with the corresponding standard deviations), depicted per instruction and per notation.

	Instructions	
Notation	Comparison	Matching
***Accuracies (% correct)***		
*Pure notations*		
digit–digit	95 (6)	97 (4)
number word–number word	94 (6)	95 (5)
dot–dot	90 (7)	94 (5)
*Mixed notations*		
number word–digit	93 (7)	98 (3)
dot–digit	93 (7)	95 (6)
number word–dot	92 (8)	95 (5)
***Reaction times (ms)***		
*Pure notations*		
digit–digit	511 (141)	535 (100)
number word–number word	530 (134)	552 (90)
dot–dot	567 (155)	578 (97)
*Mixed notations*		
number word–digit	542 (138)	546 (85)
dot–digit	562 (154)	573 (93)
number word–dot	583 (169)	584 (99)

Second, to examine whether we could replicate the finding of Lyons et al. [22], and whether they can be generalized to matching instructions, the cost for switching between symbolic and non-symbolic notations was calculated similarly as was done in the original study. Namely, Lyons et al. [22] calculated the switch cost as the difference between the RT in the mixed condition and the RT in the pure condition that yielded the worst performance. For instance, the switch cost for the digit–dot condition was computed by calculating the difference between the RT in the digit–dot condition and the RT in the dot–dot condition, because the dot–dot condition was the slowest pure one (i.e., slower than the digit–digit condition). We did exactly the same for all conditions: from RT in the mixed number word–dot condition we subtracted the RT of the dot–dot condition, and form the RT in the mixed number word–digit condition we subtracted the RT of the number word–number word condition, because these were the slowest pure conditions, respectively (see Table 1). Next, these differences between the RT in the mixed and in the pure conditions differences were submitted to an one-sample *t-*test, to check whether they significantly differed from zero. According to Lyons et al.`s [22] hypothesis, this cost should be larger than zero for differences between the digit–dot and the dot–dot condition, for the difference between number word–dot to the dot–dot condition, but not for the difference between number word–digit and number word–number word.

*The ANOVA on the accuracies* showed a significant main effect of instruction, *F*(1,35) = 21.305, *p* < 0.001, *η*_*p*_^*2*^ = 0.378, with participants performing more accurately in the matching instruction condition than in the comparison instruction condition. There was also a significant main effect of notation, *F*(5,175) = 6.963, *p* < 0.001, *η*_*p*_^*2*^ = 0.166, showing better performance in trials containing only symbolic quantities (digit–digit, number word–number word, and number word–digit). The interaction between task and notation was not significant, *F*(3.739,130.855) = 1.370, *pGG* = 0.250, *η*_*p*_^*2*^ = 0.038

*The ANOVA on the reaction times* showed a significant main effect of notation, *F*(3.625,126.872) = 20.724, *pGG* < 0.001, *η*_*p*_^*2*^ = 0.372, showing faster responses for trials with symbolic numbers (see Table 1). There was no main effect of instructions, *F*(1,35) = 0.373, *p* = 0.545, *η*_*p*_^*2*^ = 0.011, nor an interaction between instruction and notation, *F*(3.834,134.181) = 0.777, *p* = 0.537, *η*_*p*_^*2*^ = 0.022

#### Switch cost calculation

**Fig 1:**
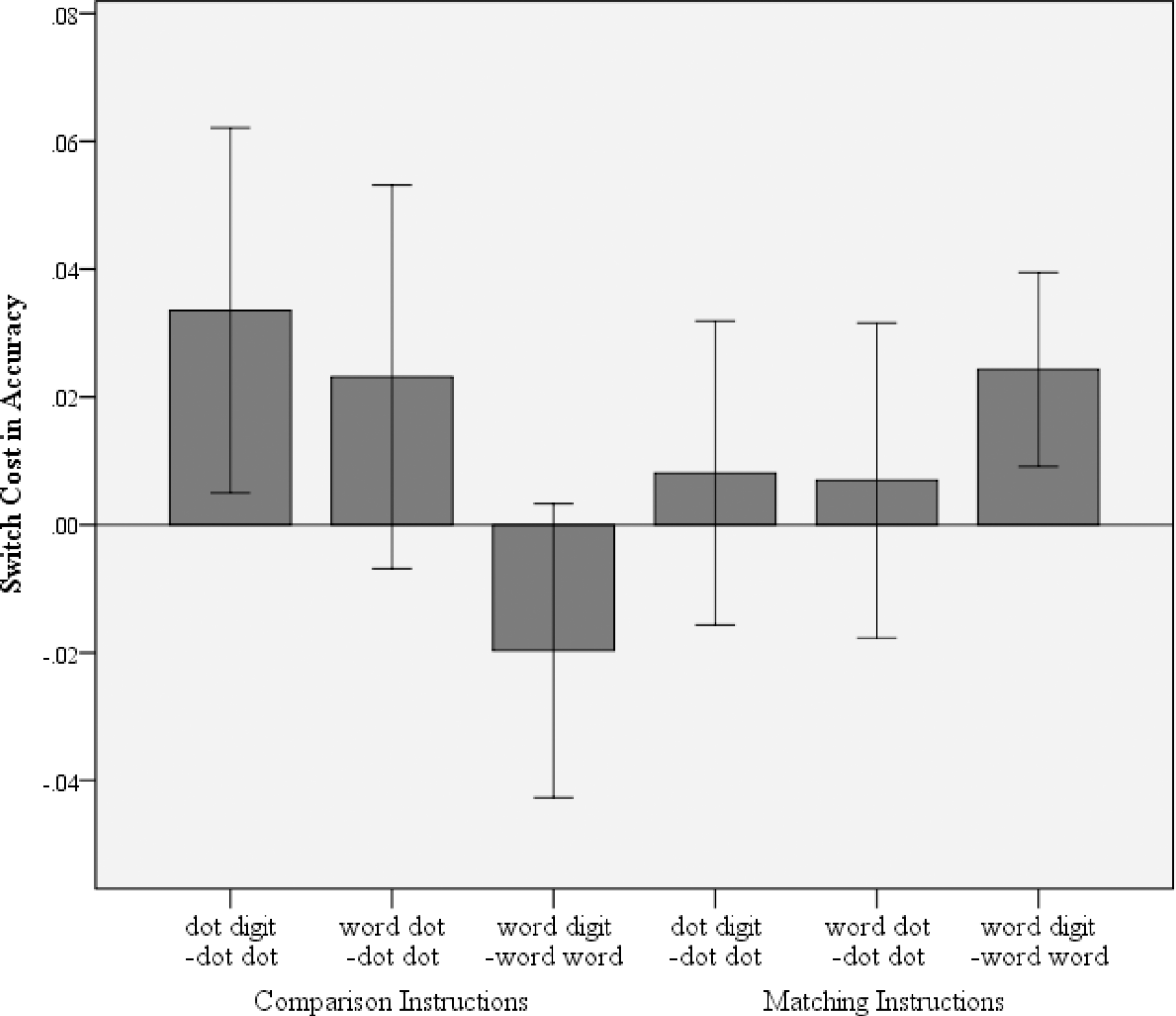
Switch cost in accuracies for both the comparison and the matching instructions in Experiment 1. Error bars denote the 95% CI.

*In the accuracies* a switch cost was present when comparing the mixed digit–dot to the dot–dot trials in the comparison condition, *t*(35) = 2.368, *p* = 0.023, *d* = 0.395. The switch cost for number word–dot to dot–dot trials was not significant *t*(35) = 1.564, *p* = 0.127, *d* = 0.261. The cost between number word–digit and number word–number was not significant also *t*(35) = –1.783, *p* = 0.091, *d* = – 0.290. In the matching instruction condition, a switch cost was present between–number word–digit and number word–number word trials, *t*(35) = 3.244, *p* = 0.003, *d* = 0.541 (see Fig 1). There was no cost for switching between digit–dot and dot–dot, *t*(35) = 0.691, *p* = 0.494, *d* = 0.115. There was also no cost between number word–dot and dot–dot trials, *t*(35) = 0.572, *p* = 0.571, *d* = 0.095.

**Fig 2:**
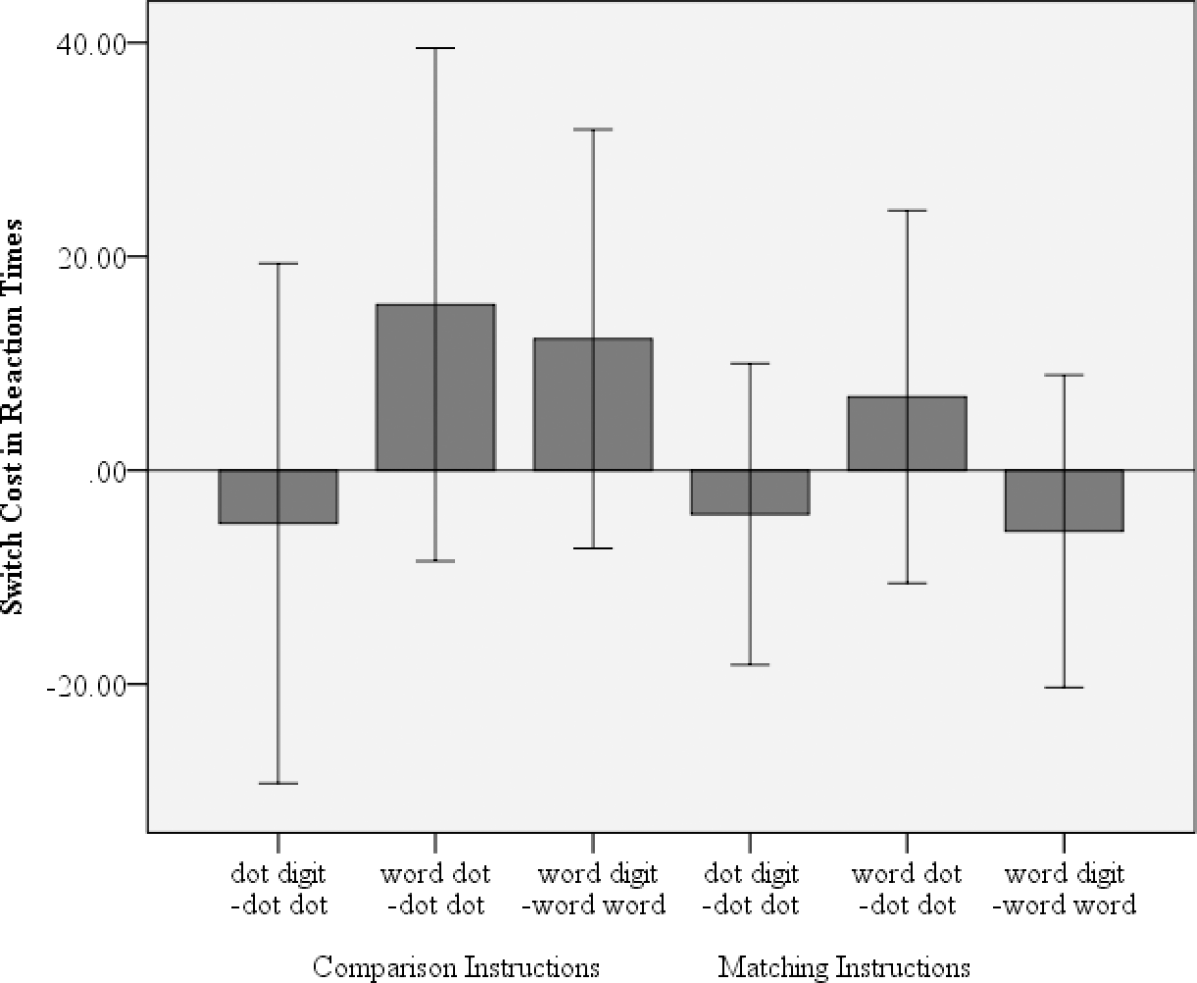
Switch cost in RTs for both the comparison and the matching instructions in Experiment 1. Error bars denote the 95% CI.

*In the reaction times* no switch costs were present in any of the instruction conditions. For the comparison condition there were no cost between digit–dot and dot–dot, *t*(35) = – 0.414, *p* = 0.681, *d* = – 0.069, between number word–dot and dot–dot, *t*(35) = 1.311, *p* = 0.198, *d* = 0.219, and between number word–digit and number word–number word, *t*(35) = 1.274, *p* = 0.211, *d* = 0.212. In the matching condition there were no costs between digit–dot and dot–dot, *t*(35) = –0.590, *p* = 0.559, *d* = – 0.098, between number word–dot and dot–dot, *t*(35) = 0.800, *p* = 0.429, *d* = 0.133, and between number word–digit and number word–number word, *t*(35) = – 0.791, *p* = 0.434, *d* = – 0.132 (see Fig 2).

### Discussion

Unexpectedly, Experiment 1 did not provide evidence for an additional processing cost in conditions where participants need to integrate symbolic and non-symbolic quantity information. Put otherwise, contrary to what we expected based on the findings reported by Lyons et al. ([22]; Experiment 2), our mixed digit–dot condition was not slower than the dot–dot condition, thus running against the hypothesis of two distinct magnitude representations, as defined by Lyons and colleagues [22]. Moreover, our results did not depend on the type of instruction, because we observed similar findings in both comparison and matching. Therefore, we reasoned that our inability to replicate the findings of Lyons et al. [22] must be due to some other methodological factors that were different between their study and our current Experiment 1. We reflect on these possible differences below.

First, in our study, the presentation of all six notation combinations was randomized, which means that participants were unaware about the format of the upcoming number pair. This uncertainty has possibly forced them to keep all notations active in the brain, in order to meet the task requirements, thus making use of cognitive control mechanisms [24,32,33]. By contrast, in the study of Lyons et al. [22], each notation combination was presented in a separate trial block. Consequently, in such a blocked design no extra cognitive effort is required, because participants know beforehand which type of trial will be presented. In other words, the blocked design applied by Lyons et al. [22] may be a more precise method for observing a switch cost, compared to a randomized design, in which other cognitive mechanisms might interfere.

Second, Lyons et al. [22] also used large numbers (10, 20, 30 and 40), while we only used small numbers that fall in the so-called subitizing range [34]. Although in the original study, a switch cost was present even for the small quantities (59ms), it was more pronounced for the larger ones (109ms) ([22] p.639). Therefore, we reasoned that the inclusion of larger numbers might enable us to detect a switch cost more easily.

Finally, Lyons et al.`s [22] instructions were slightly different from the ones we used in Experiment 1. Lyons and colleagues [22] instructed participants to decide whether the first or the second quantity was larger, or whether they were the same, whereas in our study, both judgments were conducted in separate blocks. Therefore, one could argue that the strategies used by our participants to solve the task were different from the strategies used by the participants in the original study.

Consequently, to examine whether these methodological changes were responsible for the different results of our study and the one conducted by Lyons and colleagues [22], we (1) presented the different trial types in separate blocks; (2) included larger numbers; and (3) applied the same instructions as in Lyons et al. [22]. In addition, we also added a random presentation (as in Experiment 1), with which to compare the blockwise presentation.

## Experiment 2: Randomized and blocked presentation

### Method

#### Participants

Twenty one students from the KU Leuven, aged between 18 and 31 (*M*_*age*_ = 20.48 years, *SD* = 3.40, 6 males), participated in exchange for course credits. All subjects had normal or corrected-to-normal vision and signed an informed consent prior to the experiment. No subjects were excluded for low accuracy score (≤50%) or too slow/fast responses (>3*SD* from the group mean). (Data available in S1 Dataset).

#### Procedure, task instructions and stimuli

All participants performed the same task as in Lyons et al. [22], i.e., they had to judge which one of the two sequentially presented quantities was larger, whether the first or the second, or they were both numerically equivalent. If the first quantity was larger, they pressed “a”, if it was the second–“p”; if they were the same–they pressed “SPACE” with both thumbs simultaneously, on an AZERTY keyboard. Only trials containing different quantities were considered target trials. Participants performed this task once in random trial presentation and once in blockwise trial presentation condition. In both presentation conditions, the stimuli were a combinations of small (1, 2, 3, 4) and large (10, 20, 30, 40) quantities, presented in the same six notation combinations as in Experiment 1 (i.e., digit–digit, number word–number word, dot–dot, number word–digit, dot–digit, number word–dot). To optimize the duration of the experimental procedure, the stimulus pairs for mixed notation conditions were presented only in one direction in both randomized and blocked condition, i.e., for dot–digit trials the first stimulus was always dot, for digit–number word trials the first stimulus was digit, and for number word–dot trials the first stimulus was number word. This rationale was based on the findings of Lyons et al. [22], who reported that “mixed-format performance did not depend on the presentation order” ([22] p. 637). All six notation combinations were presented completely random (cf. Experiment 1) in the randomized presentation task, whereas in the blockwise presentation task condition, each block contained only one of the possible combinations of notations, (e.g., one block only digit–digit, one block only mixed digit–dot, etc.). The presentation order and the order of the different blocks in the blocked condition were counterbalanced. Each participant was presented with 48 target and 16 filler trials, per notation. This resulted in 288 target and 96 filler trials, per presentation condition. A break was given between each 64 trials in the randomized presentation condition. Similarly, in the blockwise condition, a break was given after each block (i.e., after 64 trials). The trial procedure was identical to Experiment 1. In the random condition, ten practice trials with feedback were given prior to the actual experiment, in the blocked condition, five practice trials with feedback were given prior to each block.

### Results

Similar analyses as in Experiment 1 were conducted. First, a repeated-measures ANOVA was conducted with presentation type (two levels: blocked vs. random), number range (two levels: small vs. large) and notation (six levels) as within subject variables. Mean accuracies and median RTs per presentation type, number range and notation are depicted in Table 2. Next, switch costs were computed for all relevant conditions. Again, based on the reasoning of Lyons et al. [22], and similar to Experiment 1, here we expected a switch cost in both accuracies and RT, when comparing the digit–dot to the dot–dot condition and when comparing the number word–dot to the dot–dot condition, but not when comparing the number word–digit to the number word–number word condition. Additionally, if the way of presentation and the number range are indeed crucial factors influencing the integration between symbolic and non-symbolic quantities, the expected switch cost should–in line with Lyons et al. [22]–definitely be observed in the blocked condition, and the size of the switch cost should be larger for the larger quantities.

**Table 2:** Mean accuracies and median reaction times (with their corresponding standard deviations) depicted per presentation type, number range and notation.

	Presentation condition			
	Random		Blocked	
Quantity range	Small	Large	Small	Large
Notation				
***Accuracies (% correct)***				
*Pure notations*				
digit–digit	97 (5)	95 (4)	97 (4)	97 (4)
number word–number word	97 (4)	97 (4)	99 (2)	98 (5)
dot–dot	90 (7)	72 (17)	91 (9)	81 (10)
*Mixed notations*				
number word–digit	97 (4)	97 (4)	98 (3)	98 (3)
dot–digit	92 (6)	68 (14)	93 (7)	74 (9)
number word–dot	94 (5)	74 (10)	94 (9)	80 (11)
***Reaction times (ms)***				
*Pure notations*				
digit–digit	641 (167)	669 (172)	545 (107)	534 (108)
number word–number word	670 (172)	694 (163)	613 (120)	588 (96)
dot–dot	739 (171)	814 (260)	746 (193)	753 (233)
*Mixed notations*				
number word–digit	682 (168)	696 (174)	622 (125)	648 (141)
dot–digit	721 (203)	877 (250)	708 (196)	812 (276)
number word–dot	812 (197)	967 (351)	801 (212)	956 (294)

*The ANOVA on the accuracies* showed a significant main effect of presentation type, *F*(1,20) = 6.919, *p* = 0.016, *η*_*p*_^*2*^ = 0.257, indicating that participants responded more accurately in the blocked than in the random presentation condition. There was also a significant main effect of number range, *F*(1,20) = 89.004, *p* < 0.001, *η*_*p*_^*2*^ = 0.871, showing a better performance for small numbers. A significant main effect of notation was also present, *F*(2.852,57.046) = 84.145, *pGG* < 0.001, *η*_*p*_^*2*^ = 0.808, yielding higher accuracies for the three conditions containing only symbolic numbers (i.e., digit–digit, number word–number word and number word–digit), compared to the conditions that contain dots. Furthermore, there was a significant interaction between presentation type and number range, *F*(1,20) = 5.003, *p* = 0.037, *η*_*p*_^*2*^ = 0.200. A post hoc paired *t-*test showed that the effect of the presentation condition was significant only in case of the large numbers, because only in the large number range the participants responded more accurately in the blocked than in the random condition, *t*(20) = 2.737, *p* = 0.013, *d* = 0.597. The interaction between number range and notation was also significant, *F*(2.384,47.690) = 41.309, *pGG* < 0.001, *η*_*p*_^*2*^ = 0.674, indicating that in all notation conditions, with the exception of the two pure symbolic condition (i.e., digit–digit, *t*(20) = 0.767, *p* = 0.452, *d* = 0.167, and number word–number word, *t*(20) = 0.824, *p* = 0.419, *d* = 0.179), participants responded faster to the smaller numbers: dot–dot, *t*(20) = 4.771, *p* < 0.001, *d* = 1.041; number word–digit, *t*(20) = −2.480, *p* = 0.022, *d* = −0.541; digit–dot, *t*(20) = 11.359, *p* < 0.001, *d* = 2.479, and number word–dot, *t*(20) = 9.745, *p* < 0.001, *d* = 2.126. The presentation × notation interaction was not significant, *F*(3.204,64.081) = 2.248, *pGG* = 0.087, *η*_*p*_^*2*^ = 0.101.

*The ANOVA on the reaction times* did not show a main effect of presentation type, *F*(1,20) = 2.305, *p* = 0.145, *η*_*p*_^*2*^ = 0.103. There was a significant main effect of number range, *F*(5,20) = 16.270, *p* <0.001, *η*_*p*_^*2*^ = 0.449, with small numbers responded faster than large ones. A significant main effect of notation was also present, *F*(2,852.57.045) = 43.902, *pGG* < 0.001, *η*_*p*_^*2*^ = 0.690: responses were faster in digit–digit trials, followed by number word–number word trials, and mixed number word–digit trials (see Table 2). There was only one significant interaction–between number range and notation, *F*(2.659,53.189) = 11.396, *pGG* < 0.001, *η*_*p*_^*2*^ = 0.363. Post hoc paired-sample *t-*tests showed that only in the digit–dot trials, *t*(20) = − 3.582, *p* = 0.002, *d* = −0.782, and in the number word–dot trials, *t*(20) = −4.774, *p* < 0.001, *d* = −1.042, participants responded faster to the smallest trials. There was no presentation × notation interaction *F*(2.744,54.875) = 2.33, *pGG* = 0.089, *η*_*p*_^*2*^ = 0.104, and no presentation × range × notation interaction *F*(2.297,45.940) = 0.851, *pGG* = 0.861, *η*_*p*_^*2*^ = 0.041.

#### Switch cost calculation

**Fig 3:**
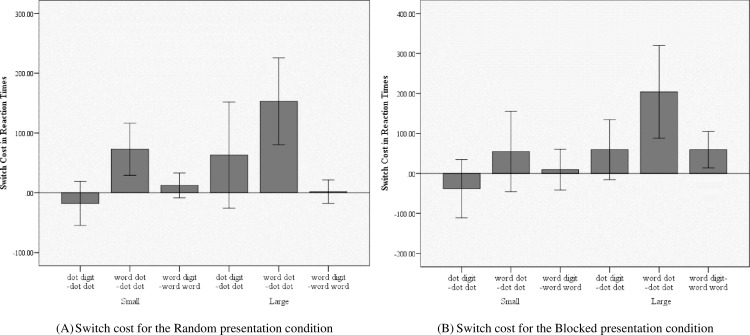
Switch cost for RTs for both small and large quantities in Experiment 2.

*Random presentation condition*. In terms of accuracies, in digit–dot trials there was significant switch cost only for small quantities, *t*(20) = 2.769, *p* = 0.012, *d* = 0.604, but not for large, *t*(20) = – 1.317, *p* = 0.203, *d* = – 0.287. In the number word–dot trials significant switch cost was present again only for small quantities, *t*(20) = 3.171, *p* = 0.005, *d* = 0.691, but not for large, *t*(20) = 0.535, *p* = 0.599, *d* = 0.117. There was no switch cost for number word–digit trials neither for small, *t*(20) = 0.213, *p* = 0.833, *d* = 0.047, or for large quantities, *t*(20) = 0.181, *p* = 0.858, *d* = 0.039. (see Fig 3). In terms of RTs, there was no switch cost for digit–dot trials neither for small, *t*(20) = –1.022, *p* = 0.319, *d* = – 0.223, or large quantities, *t*(20) = 1.477, *p* = 0.155, *d* = 0.322. In the the number word–dot trials, there was a significant switch cost for both small, *t*(20) = 4.384, *p* < 0.001, *d* = 0.957, and large *t*(20) = 3.487, *p =* 0.002, *d* = 0.760, quantities. For number word–digit trials there was no switch cost for the small, *t*(20) = 1.224, *p* = 0.235, *d* = 0.267, or large quantities, *t*(20) = 0.193, *p* = 0.849, *d* = 0.042 (Fig 3A).

*Blocked presentation condition*. In terms of accuracies, there was significant switch cost in the digit–dot condition for large quantities *t*(20) = −2.856, *p =* 0.010, *d* = −0.623 [35], but not for small, *t*(20) = 1.451, *p* = 0.162, *d* = 0.317. There was no switch cost in number word–dot trials for both small, *t*(20) = 1.267, *p* = 0.220, *d* = 0.276, and large quantities, *t*(20) = – 0.242, *p* = 0.811, *d* = 0.053. No switch cost was present in number word–digit trials for small, *t*(20) = 1.451, *p* = 0.162, *d* = 0.317, and large quantities, *t*(20) < 0.001, *p* = 1.00, *d* < 0.As for the RTs, there was no switch cost in digit–dot trials for both small, *t*(20) = – 1.087, *p* = 0.290, *d* = – 0.237, and large quantities, *t*(20) = 1.655, *p* = 0.114, *d* = 0.361. A switch cost was not present in the number word–dot condition, for small quantities, *t*(20) = 1.145, *p =* 0.266, *d* = 0.250, but a significant switch cost was present in case of the large quantities, *t*(20) = 3.672, *p =* 0.002, *d* = 0.801. There was no switch cost in the number word–digit trials for small quantities, *t*(20) = 0.383, *p =* 0.706, *d* = 0.084, but there was a switch cost in case of the large quantities, *t*(20) = 2.713, *p* = 0.013, *d =* 0.592. (Fig 3B).

(A) Switch cost for the Random presentation condition. Error bars denote the 95% CI. (B). Switch cost for the Blocked presentation condition. Error bars denote the 95% CI.

### Discussion

As was the case with Experiment 1, Experiment 2 did not provide us with consistent evidence for the presence of two distinct representations for symbolic and non-symbolic numbers. Put otherwise, in contrast to what had been observed by Lyons et al. [22], our second experiment showed an inconsistent pattern with respect to the presence of a switch cost in the mixed notation conditions, i.e., where an integration between symbolic and non-symbolic quantities was required (e.g., digit–dot and dot–number word). Additionally, these findings clearly suggest that the presence of such a switch cost is not influenced by the presentation conditions (i.e., random vs. blockwise), nor by the number range (i.e., small vs. large numbers), as we initially hypothesized, because the results remained quite similar across manipulations.

Following the rationale of Lyons et al. [22] for defining a switch cost (i.e., that the performance in the mixed condition should be worse than the performance in the most difficult pure condition), the interim conclusion of our results is that they do not provide evidence for the existence of separate magnitude systems for processing symbolic and non-symbolic numbers. Alternatively however, the lack of a consistent switch cost pattern might possibly be due to the influence of an extraneous variable, which was overlooked. Specifically, after visual observation of the RTs in both our experiments, and the RTs reported in Lyons et al. [22], we noticed that the RTs in our pure symbolic conditions were much faster than the RTs in our pure non-symbolic conditions, which was not the case in the study of Lyons et al. [22]. Clearly, such an observation might be due to a noise in the data, which can have serious consequences for the interpretation of our results. Therefore, we decided to further examine in more detail the data of the mixed conditions. More specifically, for the mixed conditions in *Experiment 1*, half of the trials consisted of a digit followed by a dot array, whereas in the other half, a dot array was followed by a digit. As a consequence of this sequential presentation, the observed RTs heavily depend on the processing time of the second stimulus, which is different for both type of trials (digit–dot vs. dot–digit). The above implies that, although a switch cost might be present in the mixed trials where a dot is followed by a digit, it might be masked by the fact that such trials will still be faster than pure dot–dot trials, simply because the second stimulus (i.e., a digit) is processed faster than a dot pattern, as also hinted by the RTs in the pure digit–digit and dot–dot conditions. To test whether these RT differences between the pure symbolic and the pure non-symbolic conditions indeed affected the interpretation of our results, we performed an additional post hoc analysis.

Following the reasoning described above, we computed the average RTs for each mixed trial type (i.e., dot–digit and digit–dot trials) separately and compared these RTs with the corresponding pure conditions. More specifically, *in Experiment 1* average RTs were computed for the comparison and matching conditions together. The mixed dot–digit trials were compared with pure digit–digit trials and the mixed digit–dot trials were compared with pure dot–dot trials. The same was done for the mixed number word–digit trials. In this way, possible differences in processing time, due to the different notations of the second stimulus, were eliminated. Therefore, we expected that the digit–dot condition would be slower than the dot–dot condition, and the dot–digit condition would be slower than the digit–digit condition. By contrast, the number word–digit condition should not be slower than the digit–digit condition, and the digit–number word condition should not be slower than the number word–number word condition. In support of our expectations, a switch cost was indeed present for the digit–dot condition, when compared with the dot–dot trials *t*(35) = 2.059, *p* = 0.047, *d* = 0.343, and for the dot–digit condition when compared with the digit–digit trials, *t*(35) = 2.258, *p* = 0.030, *d* = 0.376. Furthermore, in line with Lyons et al. [22], when both notations were symbolic, there was no switch cost: neither when the digit–number word condition was compared with the number word–number word trials, *t*(35) = 0.648, *p* = 0.521, *d* = 0.108, nor when the number word–digit was compared with the digit–digit trials, *t*(35) = 1.881, *p* = 0.068, *d* = 0.314.

*For Experiment 2*, the same analysis was applied–the average RTs for each of the mixed trials were aggregated over random and blocked trials. Because in this second experiment the mixed trials were always presented in the same order, i.e., dot–digit and digit–number word respectively (see section Procedure, task instructions and stimuli for Experiment 2), the second stimulus is always either a digit or a number word. Therefore, unlike Experiment 1, here the mixed trials for Experiment 2 were compared only with the digit–digit and number word–number word trials. Nevertheless, the results were in line with the results from Experiment 1, i.e., the dot–digit trials were significantly slower than the digit–digit trials, *t =* 7.696, *p* < 0.001, *d =* 1.679, and more importantly, the digit–number word trials were not slower than the number word–number word trails, *t =* 1.870, *p =* 0.076, *d =* 0.408. Additionally, the switch cost for dot–digit condition was significantly larger compared to the digit–number word condition, (183 ms vs. 21 ms, respectively), *t*(20) = 6.319, *p* < 0.001, *d* = 1.379.

With respect to the dot–digit switch cost, Lyons et al. [22] noted that across their three experiments, the cost was significantly larger for large compared to small quantities. Therefore, in our post hoc analysis we also examined whether the size of the switch cost differed between small and large quantities. In line with Lyons et al. [22], a paired *t-*tests for the dot–digit condition showed that the cost was significantly larger for large quantities than for small quantities (244 ms vs. 121 ms, see also Table 2), *t*(20) = 3.829, *p* < 0.001, *d* = 0.835.

Overall, this additional post hoc analysis suggests that there is a cost for switching between symbolic and non-symbolic number representations, but that this cost can be masked by the different processing times of the second stimulus due to different notations. However, these notational differences are difficult to overcome with visual presentation techniques. It seems a well-established fact that digits are generally processed faster than dot patterns (e.g., [36,37], see also [38], and experiments 1 and 2 from the current study). One way to avoid this is by using other paradigms that circumvent the problem, like audio-visual presentation (e.g., [39,14]). For example, Sasanguie et al. [14] examined the performance of adults in four different numerical audiovisual matching task conditions. In those conditions, first an auditory number stimulus was presented, followed by a visual number stimulus, and participants had to decide whether both stimuli were numerically equivalent. By orthogonal manipulation of the type of stimulus (non-symbolic versus symbolic), four conditions were created: (1) an auditory number word–visual digit condition, (2) an auditory number word–visual dot array condition, (3) an auditory tone sequence–visual digit condition and (4) an auditory tone sequence–visual dot array condition. These four conditions can be categorized into two pure conditions (i.e., pure symbolic: number word–digit and pure non-symbolic: tones–dots) and two mixed conditions (i.e., tones–digit and number word–dots). These two new categories–pure and mixed, can then be compared with each other. By collapsing the initial four experimental tasks into pure and mixed, possible differences in processing time of the second stimulus are accounted for, which was the main objective in the post hoc analysis reported above. For instance, in this audio-visual design, the dots are always preceded either by tones (when presented in the pure condition) or by number words (when presented the mixed condition). Similarly, digits are always preceded by number words (in pure condition) or by tones (in mixed condition). Therefore, participants have to respond to exactly the same stimuli in the collapsed pure and the collapsed mixed conditions. In addition, when using such a design, in contrast to Lyons et al. [22], a notation switch is also present in all pure conditions (i.e., non-symbolic dot array to non-symbolic tone sequence; symbolic visual digit to symbolic auditory number word), ensuring that RT differences between pure and mixed conditions can only be due to the integration of non-symbolic and symbolic numbers. Hence, when using audiovisual presentation to address our research question, we would expect that mixed trials are processed slower than pure trials, indicating a switch cost between symbolic and non-symbolic number representations.

Given the abovementioned advantages of the audio-visual paradigm, we conducted a final experiment adopting the audio-visual matching paradigm of the study by Sasanguie et al. [14]. In our third experiment, we replicated that study, but used comparison instructions instead of matching instructions. Moreover, at the same time we also reanalyzed the data from the original study of Sasanguie et al. [14] with the matching instructions (i.e., Experiment 1 only) and evaluated, as in the Experiment 1 of the current study, whether the cost to switch from symbolic to non-symbolic number notations is possibly affected by the type of instruction (i.e., matching vs. comparison).

## Experiment 3: Audiovisual Comparison task

### Method

#### Participants

Thirty participants were tested. Data of three of them were removed because they performed too slow/fast (> 3*SD* above or below the group mean per task condition), or made too many errors (≥ 50%). The reported analyses were performed on the remaining sample of 27 participants, aged between 18 to 50 years (*M*_*age*_ = 28.63 years, *SD* = 8.74, 11 males). (Data available in S1 Dataset).

#### Procedure, task instructions and stimuli

**Fig 4:**
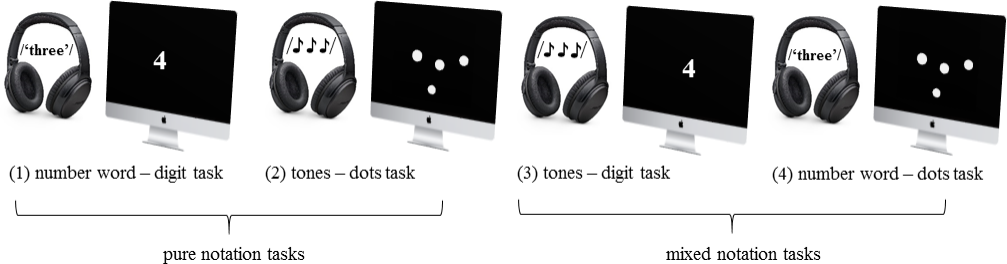
Schematic presentation of the four audio-visual tasks and their division in pure and mixed, used in Experiment 3.

The procedure, task design and stimuli set were identical to Experiment 1 of Sasanguie et al. [14], except that the participants had to make a comparison decision, instead of matching. Namely, all participants performed four audio-visual comparison tasks: (1) a number word–digit task, (2) a tones–dots task, (3) a tones–digit task and (4)a number word–dots task, which were grouped according to their notation in pure (i.e., number word–digit and tones–dot) and mixed (i.e., number word–dot and tones–digit). Stimuli consisted of the quantities 2, 3, 4, 5, 7 and 9, presented as either digits or dot arrays in the visual modality, and as spoken number words or tones (i.e., beep sequences) in the auditory modality (see Fig 4). All auditory stimuli had approximately the same duration of 1000ms. They were digitally recorded (sampling rate 44.1 kHz, 16-bit quantization) by a female Dutch speaker. The stimuli were band-pass filtered (180−10.000 kHz), resampled at 22.05 kHz and matched for loudness. The sounds were presented binaurally through headphones at about 65dB SPL. The quantities were divided in two groups as a function of their ratio (on 0–1 scale): small ratios (0.50–0.56) and large ratios (0.75–0.78) for both quantities within and outside the subitizing range (2−3 and 3−4 vs. 5−9 and 7−9). In all task conditions, first the auditory stimulus was presented, followed by the visual one. Participants had to judge which presented quantity (the auditory or the visual) was larger by pressing “a” (covered with a sticker of a microphone) and “p” (covered with a sticker of an eye) buttons on an AZERTY keyboard. Each trial began with a 600ms white fixation cross in the center of a black screen. Then the auditory stimulus was presented for 1000ms, after which the visual stimulus was presented for 1000ms. Afterwards, a blank screen was presented until response. The next trial began after a 1500ms intertrial interval. In the tones–digit and tones–dot conditions, a hissing noise was presented for 1000ms to draw participant’s attention to the whole length of the stimulus sequence. Moreover, in these conditions, high and low pitches were randomly interspersed and the intertone interval was varied randomly. Prior to each audio-visual task condition, subjects received 5 practice trials, during which feedback was provided, followed by 40 randomly presented trials without feedback. The order of the task conditions was counterbalanced across participants.

### Results

First, for replication purposes, identical and more detailed analyses per task, as those performed by Sasanguie and colleagues [14] (Ex.1), were applied to the data from this experiment. These results are reported in S1 Appendix.

Second, considering our research question about pure versus mixed tasks performance, we conducted an overall repeated measures ANOVA with task notation (pure vs. mixed), ratio (small vs. large), and number range (within subitizng vs. outside subitizing) as within subject variables. Mean accuracies and median reaction times are reported in Table 3.

**Table 3:** Mean accuracies and median reaction times (with corresponding standard deviations) in Experiment 3, depicted per task, number range and ratio.

	Quantities within the subitizing range		Quantities outside the subitizing range	
	Small ratio (0.50)	Large ratio (0.75)	Small ratio (0.56)	Large ratio (0.78)
	Trials 2–4	Trials 3–4	Trials 5–9	Trials 7–9
Comparison tasks				
***Accuracy (% correct)***				
*Pure notation tasks*	94 (7)	89 (9)	93 (9)	85 (8)
number word–digit	95 (6)	93 (9)	94 (11)	94 (11)
tones–dots	92 (14)	83 (14)	92 (11)	77 (13)
*Mixed notation tasks*	91 (9)	88 (10)	94 (8)	88 (9)
tones–digit	92 (11)	84 (17)	93 (10)	84 (15)
number word–dots	90 (12)	92 (10)	96 (10)	92 (11)
***Reaction times (ms)***				
*Pure notation tasks*	859 (255)	885 (210)	790 (227)	816 (217)
number word–digit	850 (283)	787 (185)	756 (218)	744 (169)
tones–dots	868 (288)	982 (302)	824 (289)	888 (333)
*Mixed notation tasks*	929 (207)	979 (266)	885 (243)	1031 (372)
tones–digit	968 (311)	1095(442)	973 (395)	1030 (481)
number word–dots	891 (227)	863 (198)	798 (251)	1033 (448)

*The ANOVA on the accuracies* only showed significant main effect of ratio, *F*(1,26) = 45.563, *p* < 0.001, *η*_*p*_^*2*^ = 0.637, indicating more accurate responses for small ratios. There were no main effects of task notation or number range, *F*(1,26) = 0.097, *p* = 0.758, *η*_*p*_^*2*^ = 0.004, *F*(1,26) = 0.029, *p* = .866, *η*_*p*_^*2*^ = 0.001, respectively. The task notation × number range interaction was only marginally significant *F*(1,26) = 4.057, *p* = 0.054, *η*_*p*_^*2*^ = 0.135. Because of the marginal significance of this interaction, we conduced also a paired sample *t-* test to further examine possible differences in the switch costs across the number ranges. Results showed that there was no switch cost neither for numbers within, *t*(26) = –1.243, *p* = 0.225, *d = –* 0.239, or outside subitizing range, *t*(26) = 1.724, *p* = 0.097, *d =* 0.332. The task × ration, and ratio × range interactions were not significant, *F*(1,26) = 1.034, *p* = .319, *η*_*p*_^*2*^ = 0.038, *F*(1,26) = 3.341, *p* = 0.079, *η*_*p*_^*2*^ = 0.114, respectively, and neither was the task notation × ratio × range interaction *F*(1,26) = 0.255, *p* = 0.618, *η*_*p*_^*2*^ = 0.010.

**Fig 5:**
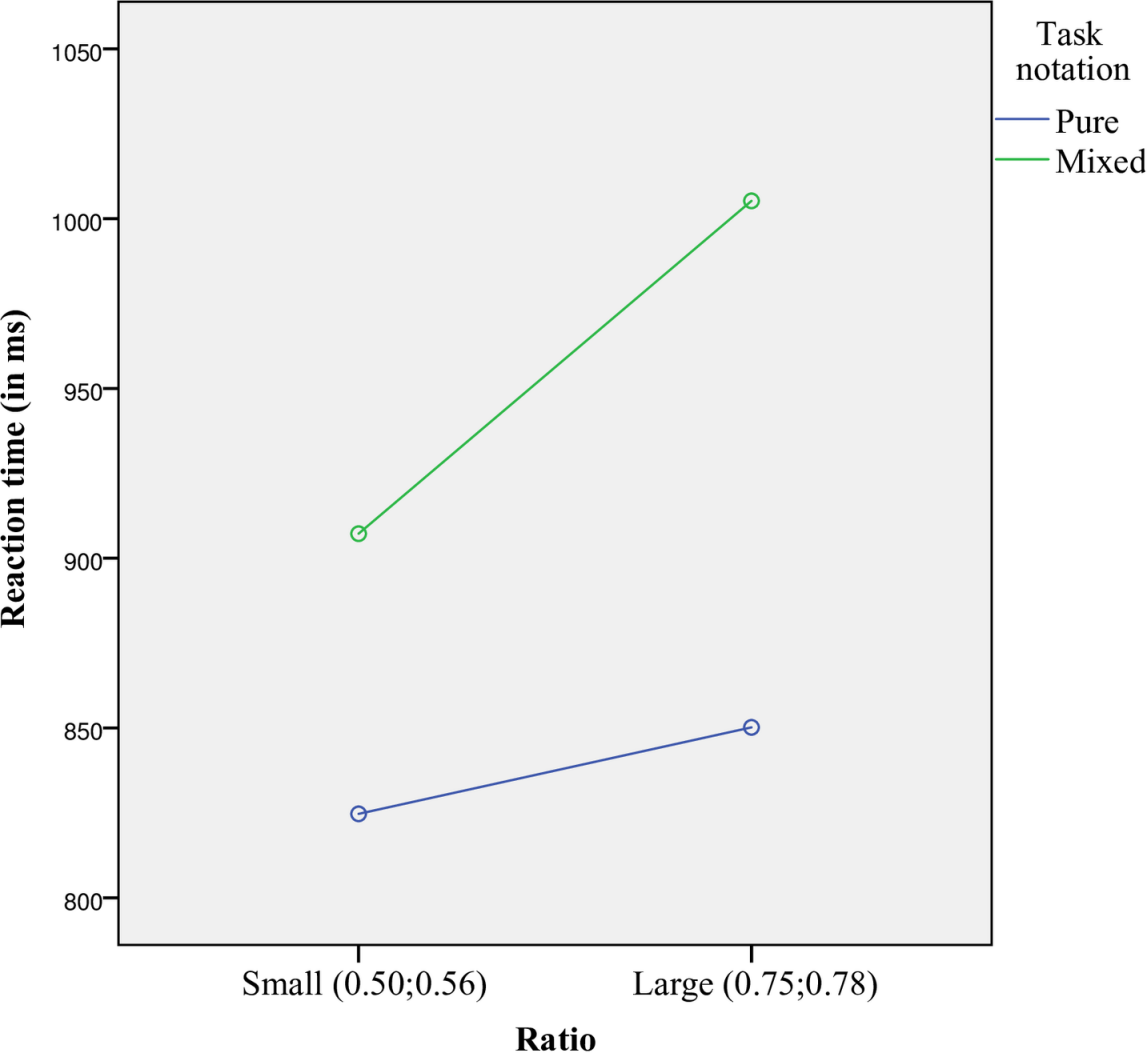
The significant task × ratio interaction.

*The ANOVA on the reaction times* showed a significant main effect of task notation, *F*(1,26) = 17.768, *p* < .001, *η*_*p*_^*2*^ = 0.406, indicating faster responses for pure notation tasks, and a significant main effect of ratio, *F*(1,26) = 15.133, *p* < 0.001, *η*_*p*_^*2*^ = 0.368, indicating faster responses for small ratios. There was no main effect of number range *F*(1,26) = 2.341, *p* = .138, *η*_*p*_^*2*^ = 0.083. The task × ratio interaction was significant, *F*(1,26) = 5.034, *p* = .034, *η*_*p*_^*2*^ = 0.162. Post hoc paired *t*-test showed that the mixed tasks were significantly slower than the pure tasks for both small and large ratios, *t*(26) = 2.829, *p* = 0.009, *d* = 0.545, and, *t*(26) = 4.367, *p* < 0.001, *d =* 0.840. Furthermore, the size of this switch cost was significantly larger for large ratios compared to small ratios, *t*(26) = 2.244, *p =* 0.034, *d* = 0.432, (155 vs. 83 ms, respectively; see Fig 5). The task × number range, and ratio × number range interactions were not significant, *F*(1,26) = 3.472, *p* = 0.074, *η*_*p*_^*2*^ = 0.118, *F*(1,26) = 2.866, *p* = 0.102, *η*_*p*_^*2*^ = 0.099, respectively, and neither was the task notation × ratio × number range interaction *F*(1,26) = 3.429, *p* = 0.075, *η*_*p*_^*2*^ = 0.117.

To examine the switch costs between pure and mixed trials in more detail, we computed the switch costs between each pure and the corresponding mixed trial, i.e., between number word–digit versus tones–digit, and number word–dots versus tones–dots, for both small and large ratios. For the number word–digit vs. tones–digit condition, significant costs were observed for both small and large ratios, *t*(26) = 3.004, *p* = 0.006, *d* = 0.578, and, *t*(26) = 4.188, *p* < 0.001, *d* = 0.806, respectively. For tones–dots vs number word–dots, there were no costs: neither for small, nor for large ratios, *t*(26) = 0.039, *p* = 0.969, *d* = 0.008, and, *t*(26) = –0.227, *p* = 0.882, *d* = –0.044, respectively. Consequently, the costs for numbers word–digit vs. tones–digit was significantly larger than the costs for tones–dots vs. number word–dots, for both small and large ratio, *t*(26) = 2.829, *p* = 0.009, *d* = 0.545, and, *t*(26) = 4.367, *p* < 0.001, *d* = 0.840, respectively.

**Fig 6:**
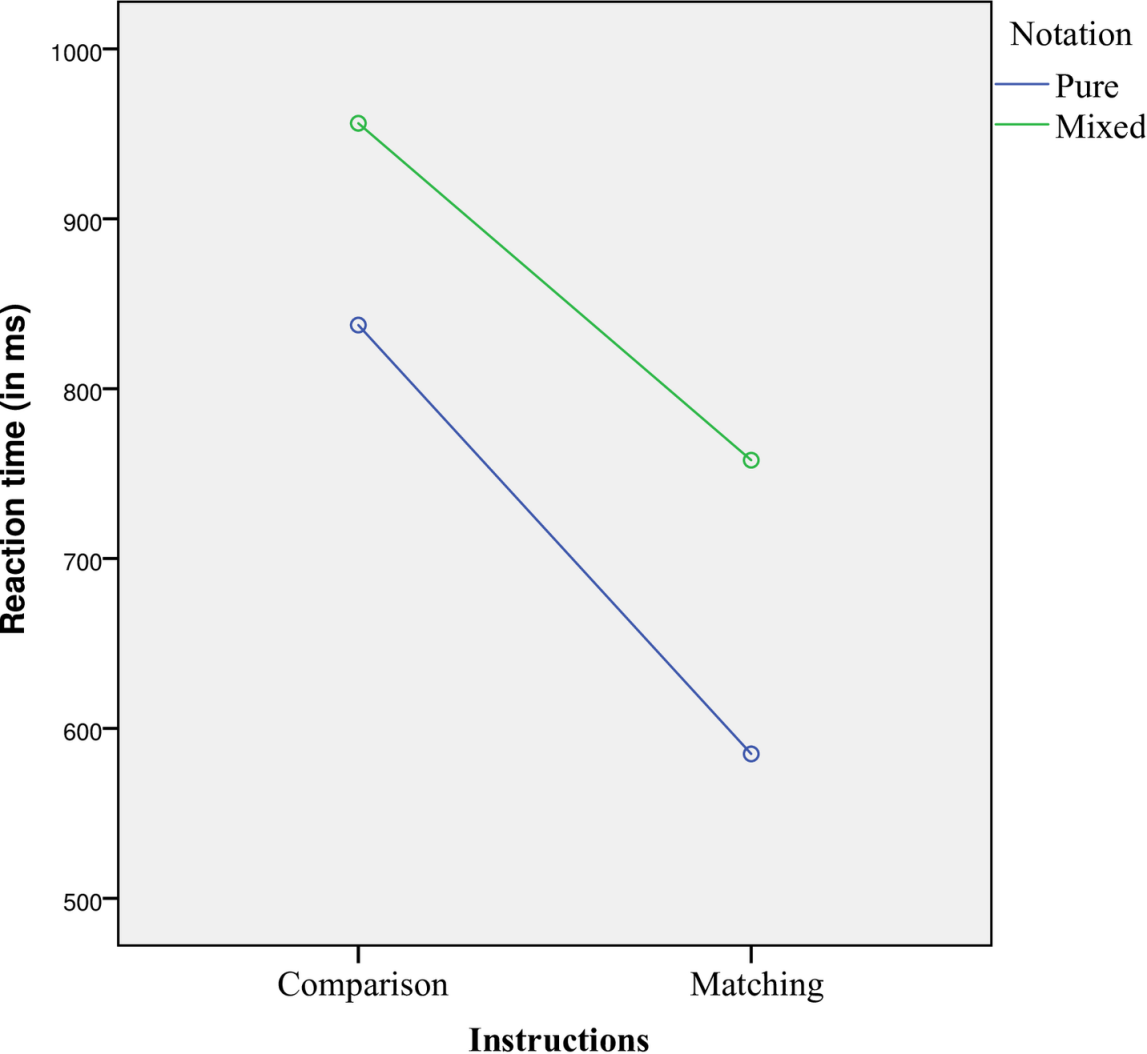
Switch costs between pure and mixed trials observed with comparison instructions (current Ex. 3), and matching instructions (Ex.1 from Sasanguie et al. [14]).

Finally, we investigated whether the switch cost, when addressed with audio-visual paradigm, is also dependent on the task instructions (i.e., comparison or matching). To this end, we compared overall switch cists of our Experiment 3 with comparison instructions with Experiment 1 of Sasanguie et al. [14] with matching instructions. For the comparison experiment, a paired sample *t-*test showed that participants indeed responded significantly slower to the mixed trials than to the pure trials, *t*(26) = 4.215, *p* < 0.001, *d* = 0.811. Similarly, for the matching experiment by Sasanguie et al. [14], the mixed trials were responded significantly slower than the pure trials, *t*(32) = 8.835, *p* < 0.001, *d* = 1.538 (see Fig 6). Taken together, these findings indicate that, when an audio-visual paradigm is applied, evidence for the dissociation between symbolic and non-symbolic numbers (in terms of a switch cost) is observed in both comparison and matching conditions.

## General discussion and conclusion

Previous findings from studies investigating whether symbolic and non-symbolic numbers are processed by one representation system or whether two distinct number systems exist, have been interpreted in a contradicting manner. Some researchers claim that there are separate systems, based on the presence of a cognitive switch cost when adults have to integrate symbolic and non-symbolic quantities [22]. Others claim that non-symbolic and symbolic numbers are automatically integrated [21]. Therefore, the aim of the present study was to use various experimental manipulations to investigate this issue again, while bridging some of the methodological differences between these previous studies.

We conducted three behavioral experiments with adults. In Experiment 1, we replicated experiments 2 and 3 of Lyons et al. [22], but instead of only using one instruction format, our participants performed the task with both comparison and matching instructions. In Experiment 2 of the current study, we examined the role of the presentation order of the trials (i.e., blocked vs. randomized presentation). At first sight, and contrary to our expectations based on the findings of Lyons et al. [22], Experiments 1 and 2 did not support the hypothesis of two distinct magnitude representation systems. However, by analyzing the data in more detail we showed that the presence of a cost when switching from symbolic to non-symbolic numbers can be masked by differences in processing times for the different notations. In order to circumvent this problem, we conducted Experiment 3, using an audio-visual paradigm. In this last experiment, as hypothesized, a clear switch cost for mixed trials was observed. Taken together, our results show that when investigating the integration between symbolic and non-symbolic quantities with a paradigm well-suited for the purpose, i.e., one that is less affected by the RT differences between symbolic and non-symbolic numbers, an additional processing cost becomes apparent. Furthermore, in line with Lyons et al.[22], the size of the switch cost was larger for large numbers in Experiment 2. For Experiment 3, the size of the switch cost was dependent on the ratios, but not on the number range–we observed larger switch costs for the more difficult ratios (0.75 and 0.78), compared to the easier ratios (0.50 and 0.56).Additionally, it should be noted that we observed a switch cost when comparing number word–digit vs. tones–digit conditions, but not when we compared tones–dots conditions vs. number word–dots. This observation is probably due to the presumed underlying representations of the presented stimuli in each condition. When a set of tones and an array of dots have to be compared, participants have to rely on two approximate representations. In contrast, when a set of tones have to be compared with a digit, an approximate representation can be compared with an exact representation. This means that, although in the former case (i.e., tones–dots) comparisons can be made within one system which should lead to faster reaction times, the fuzziness of the two approximate representations eliminates this advantage. This confound is clearly present when comparing two single conditions (e.g., number word–dots vs. tones–dots but also number word–digit vs. tones–digit), but can be eliminated when we combine the two pure conditions and the two mixed conditions, as we did in the main analyses of our Experiment 3. In this way, the number of exact and approximate representations needed to compare the stimuli in the different conditions is matched and the reaction time difference that remains between pure and mixed conditions can only be subscribed to switching from one to another system. This is clearly a big advantage of the innovative design that was presented here.

From a methodological point of view, our results show that, when comparing different types of sequentially presented trials, it is crucial to keep the second stimulus identical, in order to control for differences in processing time. The audiovisual paradigm is not affected by the above–it has been used before by Sasanguie et al. [14] and in our Experiment 3. In addition, this paradigm has several other advantages. The first one is that participants cannot base their decisions solely on the visual similarities between the stimulus properties–a problem that has been previously reported in studies using other paradigms (e.g.,[29,31,40,41,42]). Second, as we already pointed out above, a notation switch is always present in all types of trials. Consequently, a difference in performance between the pure and mixed trials is never confounded by the fact that participants have to switch between notations in mixed trials (e.g., digit–dot) but not in pure trials (e.g., dot–dot; see Experiment 1–2, Lyons et al.[22]), and is therefore most likely due to switching between underlying mental representations. Finally, the audio-visual paradigm is very well-suited to investigations of the developmental trajectory of the integration between symbolic and non-symbolic quantities, because it does not require that the participants can read (e.g., number words; for similar reasoning, see [43]).

From a theoretical point of view, our overall results are compatible with the findings of Lyons et al. [22], suggesting that symbolic and non-symbolic quantities are processed by two distinct magnitude representation systems. The question now, however, is how do these two systems look like? One suggestion regarding the features of these systems comes for the study of Sasanguie et al. [14]. There the authors argued that non-symbolic quantities are processed by the approximate system, whereas symbolic quantities are processed independently by a discrete and precise system (see also [5,19,44]). This proposal was based on the finding that a ratio effect, i.e., an effect indicating the ANS was activated, was present in all tasks containing a non-symbolic element, but was absent in the number word–digit matching task (i.e., the pure symbolic task).To account for these findings with symbolic quantities, it has recently been proposed that the *advanced* symbolic number system (i.e., such as adults have) may be considered as an associative system, consisting of relations between numerals that are formed on the basis of co-occurrences of numerals (e.g., as is the case in the counting list, adding by twos, multiplication tables, etc.). As a consequence, the association between numerals is a function of a person`s experience with symbols during his/her lifespan (e.g., [19,20,45])

However, even if there are distinct symbolic and non-symbolic representation systems, as our data indicate, interactions between the two systems remain, of course, possible. Examples of the latter are cases in which the number of dots in a dot array needs to be estimated (e.g.,[46]), or if the task instructions require that both types of quantities are compared, as was the case in the current study. A transcranial magnetic stimulation (TMS) study of Sasanguie et al. [18] suggested that the symbolic–non-symbolic integration process most likely takes place in the intraparietal sulcus (IPS). More specifically, the integration between non-symbolic and symbolic quantities in a priming task was interfered after IPS stimulation. In contrast, no interference was observed after the integration of two symbolic quantities after IPS stimulation, indicating that these symbolic quantities might be processed elsewhere in the brain. However, more research is required to further investigate the relevant brain areas and the dynamics of this integration process between non-symbolic and symbolic quantities.

In conclusion, the goal of the present study was twofold: 1) to test for the existence of separate symbolic and non-symbolic number representation systems, and 2) to investigate whether the extent to which the evidence for distinct quantity systems is found depends on the task instructions and/or other methodological factors. Taken together, the results provided evidence in favor of the existence of two separate systems, independently of the task instructions and/or presentation order. More importantly, however, we showed that this evidence can be subject to methodological drawbacks, namely, when differences between processing times for different notations are not taken into account, which can mask the cost for switching between the two systems. Therefore, future studies should consider using designs which prevent or are not affected by such pitfalls. The audio-visual paradigm we applied here is promising in this regard.

## Supporting information

S1 Dataset Data from Experiments 1–3. (ZIP)

S1 Appendix Re-analysis of the data of Experiment 1 of Sasanguie et al. [14] as a function of the research question addressed in Experiment 3 of the current study. (DOCX)
