# Supplementary material for: Symbolic estrangement or symbolic integration of numerals with quantities: Methodological pitfalls and a possible solution
Source: PLoS One. 2018 Jul 16;13(7):e0200808. doi: 10.1371/journal.pone.0200808 (PMC6047813; doi:10.1371/journal.pone.0200808)
Supplement: S1 Appendix — (DOCX) [file pone.0200808.s002.docx]

**Re-analysis of the data of Experiment 1 of Sasanguie et al. [14] as a function of the research question addressed in Experiment 3 of the current study.**

Mean accuracy scores and median reaction times (RT) on correct responses were submitted to a repeated measures analyses of variance (ANOVA) with audio-visual comparison task condition (four levels: number word – digit, tones – digit, number word – toned, number word – dots, digit – tone, dots – word, dots – tones), ratio (two levels: small vs. large) and number range (two levels: within vs. outside subitizing range) as within-subjects variables. As our research question focuses on the differences between symbolic vs. non-symbolic stimuli, post hoc ANOVAs per task condition were performed, when relevant (and post hoc analyses whenever needed).The mean accuracies and median RTs for each audio-visual comparison task, per ratio and per number range, are shown in Table 3.

*For the accuracies ,* overall ANOVA showed a main effect of task, *F*(2.220,57.726) = 9.206, *pGG* < 0.001, *η_p_^2^* = 0.261, with participants being more accurate in the digit – word task, followed by the dot – word, dot – tone and the digit – tones task. There was also a main effect of ratio, *F*(1,26) = 45.563, *p* < 0.001, *η_p_^2^* = 0.637, demonstrating that participants performed better on smaller ratios. There was a significant task × ratio interaction, *F*(3,78) = 8.380, *p* < 0.001, *η_p_^2^* = 0.244.

Post hoc ANOVAs for the *number word – digit comparison task* showed that there was no effect of ratio, no effect of number range (all *ps* < 0.05), and no interaction between ratio and range (*p* > 0.05).

In the *number word – dots comparison task*, no main effects were significant, but there was a ratio × range interaction, *F*(1,26) = 4.467 *p =* 0.044, *η_p_^2^* = 0.146. Post hoc paired-sample *t-*tests showed no significant ratio effect neither for quantities within, nor outside the subitizing range (all *ps* > 0.05).

In the *tones-digit comparison task,* there was a main effect of ratio, *F*(1,26) = 13.288, *p =* 0.001, *η_p_^2^* = 0.338, showing that participants responded more accurately for smaller ratios. There was no main effect of number range and no interaction (all *ps* > 0.05).

In the *tones-dots comparison task,* there was a main effect of ratio, *F*(1,26) = 27.579, *p* < 0.001, *η_p_^2^* = 0.514, indicating that participants performance was better on the smaller ratios. There was no main effect of number range and no interaction (all *ps* > 0.05).

*For the reaction times,* overall ANOVA showed a main effect of the task, *F*(2.037,52.950) = 6.063, *pGG =* 0.004, *η_p_^2^* = 0.189, with shortest reaction times for the number word – digit task and longest RT for the digit – tones comparison task. There was also a main effect of ratio, *F*(1,26) = 15.133, *p =* 0.001, *η_p_^2^* = 0.368, showing faster RT for smaller ratios. There was no effect of number range. There was an interaction between task and ratio, *F*(3,78) = 5.321, *p =* 0.002 , *η_p_^2^* = 0.170, which was further embedded in a significant three-way interaction between task, ratio and number range, *F*(3,78) = 6.869, *p* < 0.001, *η_p_^2^* = 0.209.

Post hoc ANOVA for the *number word – digit comparison task* showed only main effect of number range, *F*(1,26) = 7.304, *p =* 0.012, *η_p_^2^* = 0.219, suggesting faster responses for quantities outside the subitizing range, compared to quantities within. There was no interaction between ratio and number range.

In the *tones – digit comparison task*, there was a main effect of ratio, *F*(1,26) = 6.123, *p =* 0.020, *η_p_^2^* = 0.191, showing faster responses for numbers of small ratio, compared to numbers of large ratio. There was no main effect of number range and no ratio × range interaction.

In the *number word – dots comparison task*, there was a main effect of ratio, *F*(1,26) = 9.428, *p =* 0.005, *η_p_^2^* = 0.266, with faster reaction time for small ratios. No main effect of number range was present. There was a ratio × range interaction, *F*(1,26) = 16.939, *p <* 0.001, *η_p_^2^* = 0.219. Post hoc paired *t-*test showed that a significant ratio effect was present for quantities outside the subitizing range, *t*(26) = −3.928, *p* = 0.001, d = −0.756.

In the *tones – dots comparison task*, a main effect of ratio was present, *F*(1,26) = 17.643, *p* < 0.001, *η_p_^2^* = 0.404, with faster performance for small ratios. There was also a main effect of number range, *F*(1,26) = 5.430, *p =* 0.028, *η_p_^2^* = 0.173, showing faster responses for numbers outside the subitizing range. There was no significant ratio × range interaction (*p* > 0.05).
